# Gene Overexpression and RNA Silencing Tools for the Genetic Manipulation of the *S*-(+)-Abscisic Acid Producing Ascomycete *Botrytis cinerea*

**DOI:** 10.3390/ijms160510301

**Published:** 2015-05-06

**Authors:** Zhong-Tao Ding, Zhi Zhang, Di Luo, Jin-Yan Zhou, Juan Zhong, Jie Yang, Liang Xiao, Dan Shu, Hong Tan

**Affiliations:** 1 Key Laboratory of Environmental and Applied Microbiology, Chengdu Institute of Biology, the Chinese Academy of Sciences, No. 9 Section 4, Renmin Nan Road, Chengdu 610041, China; E-Mails: dzhongtaochina@163.com (Z.-T.D.); zhangzhi_asia@163.com (Z.Z.); lddd24@163.com (D.L.); zhoujy@cib.ac.cn (J.-Y.Z.); zhongjuan@cib.ac.cn (J.Z.); yangjie@cib.ac.cn (J.Y.); xiaoliang@cib.ac.cn (L.X.); 2 University of the Chinese Academy of Sciences, No. 19A Yuquan Road, Beijing 100049, China

**Keywords:** *Botrytis cinerea*, gene overexpression, RNA silencing, ATMT

## Abstract

The phytopathogenic ascomycete *Botrytis cinerea* produces several secondary metabolites that have biotechnical significance and has been particularly used for *S*-(+)-abscisic acid production at the industrial scale. To manipulate the expression levels of specific secondary metabolite biosynthetic genes of *B. cinerea* with *Agrobacterium tumefaciens*-mediated transformation system, two expression vectors (pCBh1 and pCBg1 with different selection markers) and one RNA silencing vector, pCBSilent1, were developed with the In-Fusion assembly method. Both expression vectors were highly effective in constitutively expressing eGFP, and pCBSilent1 effectively silenced the eGFP gene in *B. cinerea*. *Bcaba4*, a gene suggested to participate in ABA biosynthesis in *B. cinerea*, was then targeted for gene overexpression and RNA silencing with these reverse genetic tools. The overexpression of *bcaba4* dramatically induced ABA formation in the *B. cinerea* wild type strain Bc-6, and the gene silencing of *bcaba4* significantly reduced ABA-production in an ABA-producing *B. cinerea* strain.

## 1. Introduction

*Botrytis cinerea* is a notorious phytopathogenic ascomycete that causes gray mold disease in more than 200 host plant species and significantly damages large amounts of pre- and postharvest crops around the world every year [1,2]. Due to the significant economic relevance, the availability of molecular tools and the genome sequences, *B. cinerea* has been the most extensively studied necrotrophic phytopathogenic fungus [3]. More than 100 genes were shown to affect virulence or pathogenicity of *B. cinerea* [4].This indicated genetic complexity of the fungus and the pathogenic mechanisms of *B. cinerea* are still far from being uncovered. However, the impact of *B. cinerea* is not always negative. The *B. cinerea* T4 and B05.10 genome sequences predicted 43 genes probably encoding key enzymes for secondary metabolite (SM) biosynthesis [5]. This fungus has also been found to synthesize several phytohormones: IAA [6], ethylene [7], cytokinins [8], and ABA [9]. These secondary metabolites and phytohormones may have potential economical value, and together with *B. cinerea* have been particularly used for the fermentative production of ABA at the industrial scale [10,11,12].

Given the significant economic relevance of *B. cinerea*, many efforts have been made to elucidate the plant-pathogen interaction mechanisms of this fungus and to investigate other *B. cinerea* genes such as those that play their roles in SM biosynthesis.

The most widely used transformation method for *B. cinerea* is the protoplast-based transformation system which was first developed by Hamada *et al.* [13] and modified by van Kan *et al.* [14]. Based on this transformation method, targeted gene inactivation via homologous recombination has provided a powerful strategy to uncover the gene functions of *B. cinerea*. This strategy, which features a high gene replacement efficiency, has been widely utilized in *B. cinerea* strains [15,16]. *Bcku70*- and *bcku80*-deficient *B. cinerea* strains that feature an increased homologous recombination frequency have also been constructed to avoid non-homologous end joining, which facilitated gene deletion manipulations for the genes hard to knock out in wild-type *B. cinerea* strains [17]. Nevertheless, this strategy is not effective for essential genes or multi-copy genes, and RNA-mediated gene silencing has emerged as an efficient genetic manipulation tool for these cases. RNA-mediated gene silencing, also known as RNA interference (RNAi), results in diminished transcription levels of the target genes and is an alternative genetic manipulation strategy in analyzing gene function. Patel *et al.* [18] first reported the RNA silencing of a superoxide dismutase gene in *B. cinerea*, and the functions of several other *B. cinerea* genes that participate in pathogenicity or other biological processes have since been investigated using RNA interference [19,20,21]. Espino *et al.* [22] compared the silencing efficiency of two strategies to generate the dsRNA constructs in *B. cinerea*, and the results showed that the hairpin RNA-expressing strategy generated a higher proportion of strongly silenced transformants than the opposing dual-promoter strategy. The protoplast-based transformation method was used in all the RNA silencing studies reported so far in *B. cinerea*, with one exception [23]. However, the protoplast preparation protocols of this transformation method are considered tedious and often varied greatly between benches [24,25,26,27].

The *Agrobacterium tumefaciens*-mediated transformation (ATMT) system, which transfers part of the tumor-inducing plasmid DNA (T-DNA) into the genomes of the recipient, was originally developed for plant species and has been successfully used to transfer foreign DNA into fungal genomes [28,29,30,31,32]. ATMT has its advantages in manipulating a fungal gene: it features a high transformation efficiency [28,30,31] and the ability to transform both hyphae and spores [28] without the need for an enzymatic treatment. Rolland *et al.* [33] first reported the ATMT of *B. cinerea* and Giesbert *et al.* [19] used ATMT to characterize new pathogenicity-related genes of *B. cinerea* via random insertion mutagenesis. Cui *et al.* [34,35] reported the disruption of a class V chitin synthase gene (*Bcchs*5) and a class VI chitin synthase gene (*Bcchs6*) of *B. cinerea* via ATMT. Rolland *et al.* [23] silenced a putative phospholipase D-encoding gene via ATMT and this is the only report that RNA silencing via this transformation method has been performed in *B. cinerea*.

In our laboratory, a series of *B. cinerea* strains has been used for ABA biosynthetic studies, including a wild type strain Bc-6 [36] and a mutant strain TB-3-H8 which was particularly used for fermentative production of ABA with the productivity of 1.4 g/L [10,12]. Novel binary vectors were developed in this study to facilitate genetic manipulation in these *B cinerea* strains using ATMT: two gene expression vectors (pCBh1 and pCBg1) with different selection markers and one RNA silencing vector pCBSilent1. An enhanced green fluorescent protein (eGFP) was used to validate the working efficiency of these genetic manipulation tools in *B. cinerea* strains. A gene *bcaba4*, suggested to participate in biosynthesis of ABA in *B. cinerea* [26], was also targeted for gene overexpression and RNA silencing with these genetic manipulation tools. These results indicated high expression efficiency or silencing efficiency of these newly developed genetic manipulation tools.

## 2. Results

### 2.1. Construction of Three Binary Vectors Suitable for the ATMT of B. cinerea Strain Bc-6

The *B. cinerea* wild type strain Bc-6 [36] was isolated from wheat stems and leaves in southwest China. Three new binary vectors suitable for the ATMT of this strain were constructed to facilitate the functional investigation and exploration of genes related to the SM biosynthesis of this phytopathogenic ascomycete. The novel *Agrobacterium* vector pCBh1 for gene overexpression was constructed based on the pBHt2 backbone and contained two cassettes in its T-DNA region: a hygromycin-resistance cassette derived from the vector pBHt2 [30] and an expression cassette, in which the MCS for insertion of targeted genes was driven by the *Aspergillus nidulans oliC* promoter and terminated by the *Aspergillus nidulans trpC* terminator. The novel expression vector pCBg1 was developed based on the backbone of the *Agrobacterium* vector pCAMBA-1300-221 (available online: http://www.cambia.org.au). The T-DNA region of pCBg1 also contains two cassettes: an expression cassette that comprises the same modules as pCBh1 and a glufosinate-resistance cassette, which consists of the *Aspergillus nidulans trpC* promoter derived from the vector pBHt2, the basta-resistance gene derived from the vector pLOB7 [37] and the CaMV35S polyA terminator derived from the vector pCAMBA-1300-221. The binary vector pCBSilent1 for gene silencing with the ATMT system was developed based on the backbone of the newly constructed expression vector pCBh1 and also contained two cassettes in its T-DNA region: the hygromycin-resistance cassette of pCBh1 and a transcriptional unit that facilitates hairpin RNA generation. The two MCS modules of the vector pCBSilent1 [38] were separated by intron 2 of the *M. oryzae* cutinase gene and were used to insert the sense and antisense sequences of targeted genes. All the three binary vectors contain the kanamycin-resistance cassette which facilitates the screening of positive *E. coli* or *A. tumefaciens* colonies.

**Figure 1:**
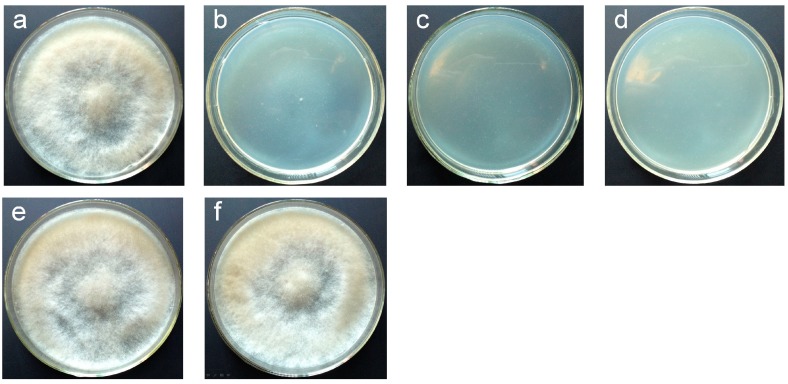
Sensitivity of *B. cinerea* wild type strain Bc-6 to glufosinate-ammonium and mitotic stability of transformants derived from ATMT using vector pCBg1. Ten μL of conidial suspension (1 × 10^6^ conidia/mL) was inoculated on potato dextrose agar (PDA) plates in the presence of (**a**) 0 μg/mL; (**b**) 15 μg/mL; (**c**) 25 μg/mL and (**d**) 50 μg/mL glufosinate-ammonium and cultured at 26 °C for 7 days; (**e**) The 1st generation transformants and (**f**) the 6th generation transformants derived from ATMT of *B. cinerea* Bc-6 using vector pCBg1 were grown on PDA medium supplemented with 25 μg/mL glufosinate-ammonium at 26 °C for 7 days.

The sensitivity of *B. cinerea* wild type strain Bc-6 to hygromycin B and glufosinate-ammonium was verified by inoculating the conidia of this fungus on PDA plates containing different concentrations of the two antibiotics. Hygromycin B (25 μg/mL) or glufosinate-ammonium (25 μg/mL) completely arrested the growth of *B. cinerea* Bc-6 (Figure 1) and these concentrations were used to select transformants in further experiments. The ATMT [19,33] of *B. cinerea* Bc-6 was performed using the vectors pCBh1, pCBg1 and pCBSilent1. Of 1 × 10^6^ conidia inoculated on PDA medium plates supplemented with 25 μg/mL hygromycin B (for pCBh1 and pCBSilent1) or 25 μg/mL glufosinate-ammonium (for pCBg1), 500–1000 transformants derived from ATMT were selected for each vector. The genetic stability of the transformants was also investigated. Six randomly selected transformants for each vector were subcultured on PDA plates without any antibiotics for five successive generations, and the sixth generation transformants were cultured on PDA plates in the presence of the same antibiotics as those used for their ancestor transformants. After incubation at 26 °C for seven days, all transformants of the sixth generation showed the same antibiotic resistance as their first generation ancestors (Figure 1), indicating that the T-DNA integration into the genome was stable during the growth and differentiation of *B. cinerea* Bc-6. These results confirmed that these new binary vectors for gene expression or RNA silencing are suitable for the ATMT of *B. cinerea* wild type strain Bc-6.

### 2.2. Vectors pCBh1 and pCBg1 Expressed eGFP in B. cinerea Bc-6 with High Efficiency

**Figure 2:**
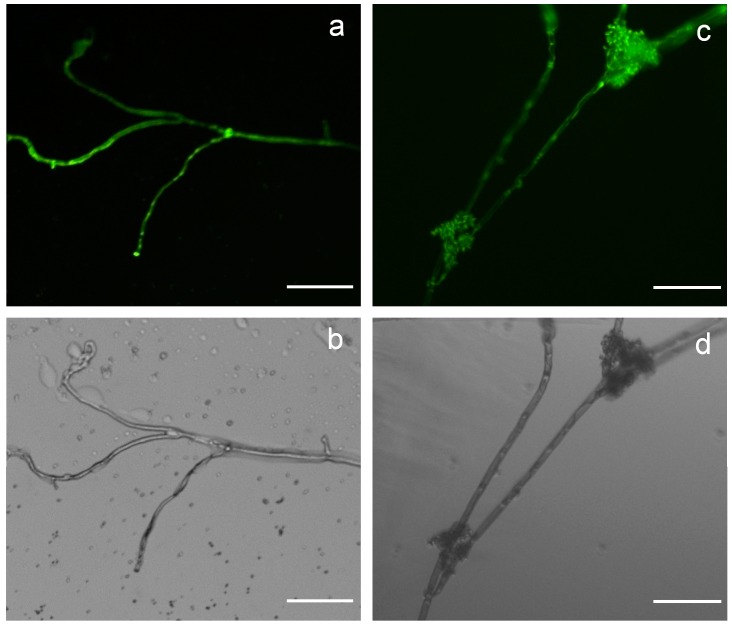
The pCBg1-eGFP vector induced GFP expression in *B. cinerea* Bc-6 with high efficiency. GFP fluorescence and bright field images of the mature hyphae (**a**,**b**) and conidia forming hyphae (**c**,**d**) of representative transformants derived from ATMT of the *B. cinerea* wild type strain, Bc-6, using the expression vector pCBg1-eGFP. Scale bar: 100 μm.

To determine the gene expression efficiency of the two novel expression vectors pCBh1 and pCBg1, an exogenous gene, *eGFP* whose expression could be easily visualized, was targeted for gene overexpression in *B. cinerea* with these vectors. The *eGFP* CDS from the vector pEGFP-N1 (Clontech, Mountain View, CA, USA) was amplified and inserted into the MCS of vectors pCBh1 and pCBg1 with In-Fusion assembly methods, and two eGFP overexpression vectors, pCBh1-eGFP and pCBg1-eGFP, were generated with hygromycin and glufosinate as the selection markers respectively. The ATMT of the conidia of *B. cinerea* Bc-6 was performed using the two eGFP expression vectors. After regenerating the single conidia of the transformants in the presence of corresponding antibiotics, DNA was extracted from 10 randomly selected hygromycin-resistant transformants that contained the vector pCBh1-eGFP and 10 randomly selected glufosinate-resistant transformants that contained the vector pCBg1-eGFP. Diagnostic PCR verification using the primer pair PAnoliC-579 and TAntrpC-132 (Table 1) confirmed that the T-DNA regions of the vectors had integrated into the genomes of all these randomly selected transformants (data not shown). The eGFP fluorescence of these randomly selected transformants was visualized on a Zeiss Axioplan 2 fluorescence microscope (Figure 2). The eGFP fluorescent images of the conidia and mature aerial hyphae of the transformants indicated that the vector pCBg1 expressed eGFP in *B.cinerea* Bc-6 with high efficiency; the same results were obtained for pCBh1 (data not shown). Because the *Aspergillus nidulans oliC* promoter used in the expression vectors is a constitutive promoter, the *eGFP* gene under the control of this promoter was expressed in all of the tissues of the transformants.

**Table 1:** The primers used in this study for vector construction, diagnostic PCR verification and RT-PCR analysis.

Primer Name	5'–3' DNA Sequence
pUC19-AnoliC	AGCTCGGTACCCGGGCTGCAGCTGTGGAGCCGCAT
pUC19-PstI-AnoliC	CTGCAGGTCGACTCTAGAGGATCCCCGGGTACCGAGCTCGAATTCGGATCGATTGTGATGTGATG
pUC19-BamHI-AntrpC	GGATCCTCTAGAGTCGACCTGCAGGCATGCAAGCTTAGTAGATGCCGACCGGGATC
pUC19-AntrpC	GCCAAGCTTGCATGCTAGGCAACCATGCATGGTTAC
p1300-AntrpC	CATGATTACGAATTCTAGGCAACCATGCATGGTTACTATTG
p1300-AnoliC	TGCCAAGCTTGCATGCTGCAGCTGTGGAGCCGCATTCCG
AnoliC-eGFP	CATCACAATCGATCCATGGTGAGCAAGGGCGAGGAGCTG
AntrpC-eGFP	CGGTCGGCATCTACTCTAGATCCGGTGGATCCCGGGC
bar-5	ATGAGCCCAGAACGACGCCCG
bar-3	ATCTACTTCAGATCTCGGTGACGGGCAGGACCGGACGGGGCGGTAGCGGCAGG
p1300-EcoRI	TATGACCATGATTACTAGGCAACCATGCATGGTTACTATTG
p1300-HidIII	ACGACGGCCAGTGCCCTGCAGCTGTGGAGCCGCATTCCG
5-AnoliC-CUT	CATCACAATCGATCCTCTAGAGGTACCGCTGGAGGATACAGG
3-AntrpC-CUT	CGGTCGGCATCTACTGAGCTCGGATCCGCCGTTCCCTGGCTG
PAntrpC-3	GGTAGAATAGGTAAGTCAGATTGAATCTG
CaMV35S-5	GGGATCTCGAGTTTCTCCATAATAATG
BstXI-PAntrpC-5	GGCTAGAGCAGCTTGCCAACATGGTGGGTCGACAGAAGATG
SacII-leftborder-3	GAGCCGATTTTGAAACCGCGGTGATCACAGGCAGCAACGC
Silent-eGFP-Se-5	CATCACAATCGATCCATGGTGAGCAAGGGCGAGG
Silent-eGFP-Se-3	CCTGTATCCTCCAGCCTTGTACAGCTCGTCCATGCCG
Silent-eGFP-AS-5	CAGCCAGGGAACGGCCTTGTACAGCTCGTCCATGCCG
Silent-eGFP-AS-3	CGGTCGGCATCTACTATGGTGAGCAAGGGCGAGG
RT-gfp-5	CACTACCTGAGCACCCAGTC
RT-gfp-3	CACGAACTCCAGCAGGACC
RT-ABA4-5	AAGACTTGGACGAGTGGGAGTT
RT-ABA4-3	CCGTTGTTAGCCATTACTTTCAG
RT-tubA-5	GCGTTCGTGCATTGGTATGT
RT-tubA-3	CACGGGCCTCAGAGAATTCA
AnoliC-ABA4	CATCACAATCGATCCATGTCCTCTCAACCATTCACGAAC
AntrpC- ABA4	CGGTCGGCATCTACTCTAACATCTCCATCCGCCATCAATGC
PAnoliC-579	GGCTTCGTACGGGAGGTTCGGCGTAG
TAntrpC-132	TCTGCTTCGCCGGAGCCTGAAGGGCG
Silent-ABA4-Se-5	CATCACAATCGATCCATGTCCTCTCAACCATTCACGAAC
Silent-ABA4-Se-3	CCTGTATCCTCCAGCACATCTCCATCCGCCATCAATGCT
Silent-ABA4-AS-5	CAGCCAGGGAACGGCACATCTCCATCCGCCATCAATGCT
Silent-ABA4-AS-3	CGGTCGGCATCTACTATGTCCTCTCAACCATTCACGAAC

The underlined sequences indicated the 15-bp homologous oligonucleotides that facilitate In-Fusion Assembly reactions.

### 2.3. Vectors pCBh1 and pCBg1 Expressed Bcaba4 in B. cinerea Bc-6 with High Efficiency

**Figure 3:**
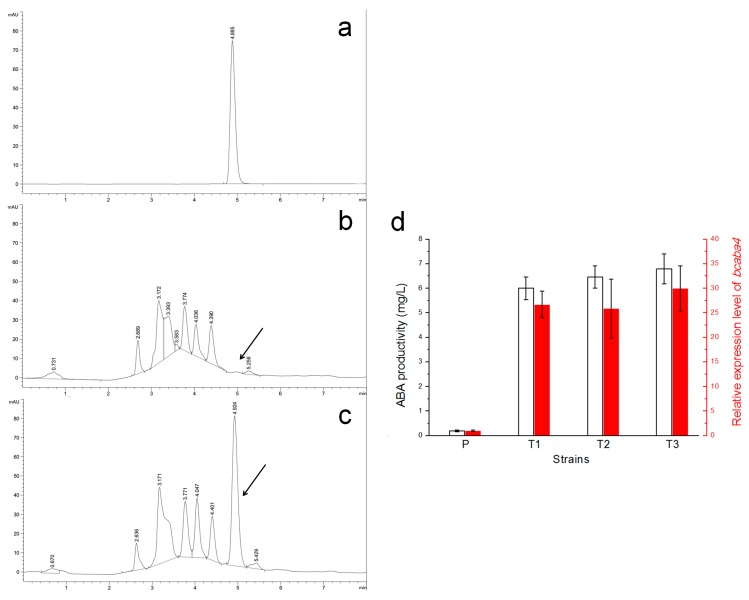
The expression vector pCBh1 expressed *bcaba4*, a SM biosynthetic gene of *B. cinerea*, with high efficiency. (**a**) A commercial *S*-(+)-ABA was used as the standard sample; (**b**) the parent strain Bc-6 showed a pretty low absorption peak corresponding to ABA; (**c**) the representative transformant showed a significantly higher absorption peak corresponding to ABA. The absorption peaks corresponding to ABA are indicated by arrows; (**d**) ABA productivity and expression levels of *bcaba4* in three randomly selected transformants are presented. The transformants were derived from ATMT of the *B. cinerea* wild type strain, Bc-6, using the expression vector pCBh1-ABA4. The expression levels of *bcaba4* were determined by qRT-PCR and presented as percentage of the value obtained from the parent strain. P: the parent strain Bc-6; T1: a randomly selected transformant whose liquid chromatogram was shown (**c**); T2, T3: two other randomly selected transformants.

After determination of the expression efficiency of vectors pCBh1 and pCBg1 with an exogenous gene, an endogenous gene, *bcaba4*, which encodes a short-chain dehydrogenase/reductase and was suggested to participate in ABA biosynthesis of *B. cinerea* strains [26], was overexpressed with the two expression vectors. The *bcaba4* CDS was amplified from the cDNA of the *B. cinerea* Bc-6 strain and inserted into the MCS of vectors pCBh1 and pCBg1 with In-Fusion assembly methods to generate two expression vectors, pCBh1-ABA4 and pCBg1-ABA4, which contained hygromycin and glufosinate selection markers respectively. The ATMT of the conidia of Bc-6 was also performed. The DNA was extracted from 10 randomly selected hygromycin-resistant transformants and 10 randomly selected glufosinate-resistant transformants, and diagnostic PCR verification was performed using the primer pair PAnoliC-579 and TAntrpC-132. The qRT-PCR results showed that the expression of *bcaba4* mRNA was dramatically increased in the randomly selected transformants compared to that of the wild type strain (Figure 3d). Single conidia of the transformants and their control strain, Bc-6, were grown on PDA plates for 15 days. Additionally, the amounts of ABA secreted by the mycelium of the transformants and their control strain were determined by HPLC using a commercial *S*-(+)-ABA as the standard sample. The absorption peak of ABA was hardly visualized when HPLC determination was performed on the control strain, Bc-6 (Figure 3b), which indicated a pretty low concentration of ABA. The results of the same assay for the transformants showed a significantly higher absorption peak corresponding to ABA (Figure 3a,c). The titers of the ABA secreted by the transformants and Bc-6 were also determined by a plant hormone abscisic acid ELISA kit. The results showed that the ABA productivity of the representative transformant showed in Figure 3c was 6.0 mg/L culture medium, which was 30 times the value determined from the parent strain (Figure 3d). The results from the three randomly selected transformants showed a correlation between their ABA productivity and their expression levels of *bcaba4* (Figure 3d). The same results were obtained for pCBg1 (data not shown).

### 2.4. Efficient Silencing of the eGFP Gene by a pCBSilent1-Based Vector

**Figure 4:**
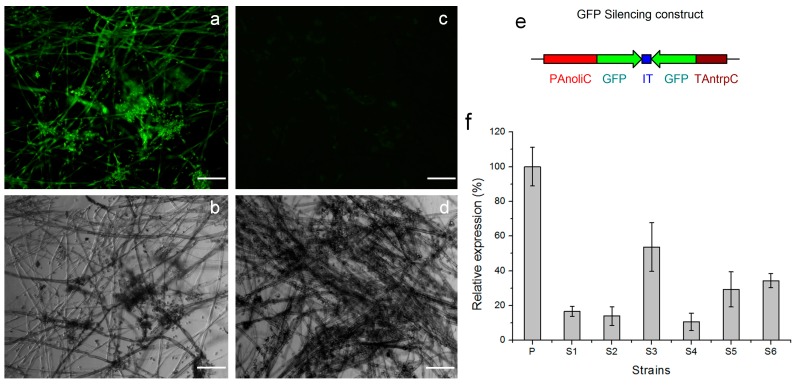
The silencing vector pCBSilent1-eGFP induced GFP silencing in *B. cinerea* with high efficiency. GFP fluorescence and bright field images of the mature hyphae of a representative transformant (**c**,**d**) derived from ATMT of the parent eGFP-expressing strain Bc-6-eGFP (**a**,**b**) were presented. Scale bar: 100 μm; (**e**) Schematic structure of the silencing construct in the vector pCBSilent1-eGFP which facilitated hairpin RNA generation; (**f**) Expression levels of *eGFP* mRNA in six randomly selected transformants were determined by qRT-PCR and presented as percentage of the value obtained from the parent strain Bc-6-eGFP. P: the parent strain Bc-6-eGFP; S1: a randomly selected transformant whose GFP fluorescence and bright field images were shown (**c**,**d**); S2–S6: five other randomly selected transformants.

To determine the silencing efficiency of the novel gene silencing vector pCBSilent1, the *eGFP* gene was targeted for gene silencing in an eGFP-expressing *B. cinerea* strain, Bc-6-eGFP, which was derived from *B. cinerea* Bc-6. The sense and antisense sequences of the 717bp *eGFP* fragment (without the termination codon) were inserted into the two MCS separated by the spacer in the vector pCBSilent1 to generate the RNA silencing vector pCBSilent1-eGFP. ATMT was performed to introduce the T-DNA region of this vector into the genome of the *B. cinerea* strain Bc-6-eGFP. After the single conidia of the transformants were regenerated in the presence of 25 μg/mL hygromycin B, DNA extraction, total RNA extraction, and cDNA synthesis were performed on 45 randomly selected transformants. Diagnostic PCR using the primer pair PAnoliC-579 and TAntrpC-132 confirmed the integration of the silencing construct into the genomes of all these randomly selected transformants. The GFP fluorescence of these transformants was visualized on a Zeiss Axioplan 2 fluorescence microscope (Figure 4). All of the 45 transformants showed varying degrees of reduced fluorescence intensity and 20 of them showed the barely detectable GFP fluorescence, similar to that shown in Figure 4c. Expression levels of *eGFP* mRNA in six of the 45 randomly selected transformants were determined by qRT-PCR (Figure 4f). *EGFP* mRNA levels in three of the six transformants were reduced to less than 20% of the value obtained from the parent strain Bc-6-eGFP, which indicated that the eGFP expression in these transformants was dramatically reduced by the silencing construct generated from the vector pCBSilent1-eGFP. These results suggested that pCBSilent1-based vectors could induce gene silencing efficiently in *B. cinerea* Bc-6.

### 2.5. Efficient Silencing of the Bcaba4 Gene in an ABA-Producing B. cinerea Mutant

**Figure 5:**
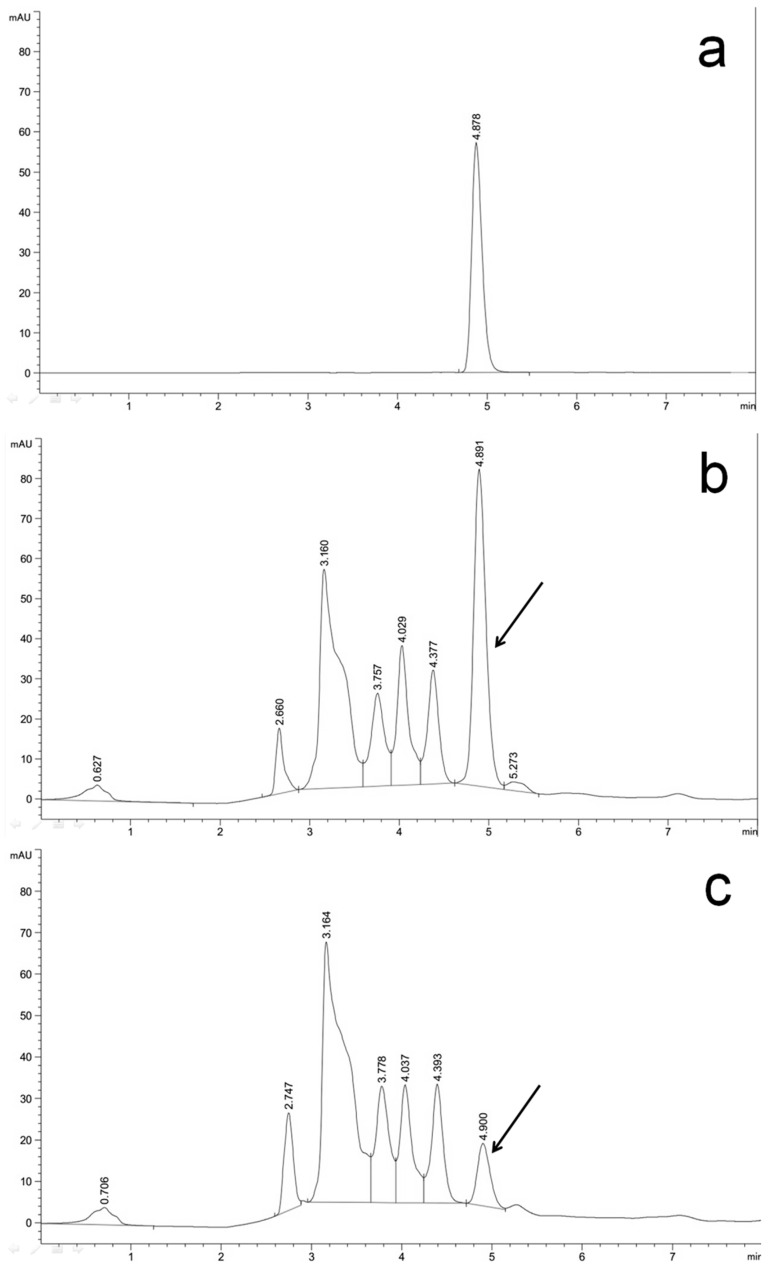

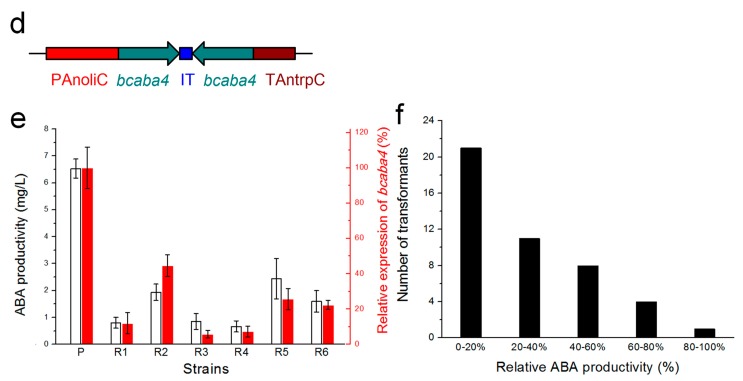
The silencing vector pCBSilent1-ABA4 silenced the *bcaba4* gene with high efficiency; (**a**) a commercial *S*-(+)-ABA was used as the standard sample; (**b**) a moderately high absorption peak corresponding to ABA was detected in the culture medium of the parent strain *B. cinerea* Bc-6-A4; (**c**) the significantly diminished absorption peak corresponding to ABA was detected in the culture medium of a representative transformant. The absorption peaks corresponding to ABA are indicated by arrows; (**d**) schematic structure of the silencing construct in the vector pCBSilent1-ABA4 which facilitated hairpin RNA generation; (**e**) ABA productivity and expression levels of *bcaba4* mRNA in six randomly selected transformants are presented. The transformants were derived from ATMT of the ABA-producing *B. cinerea* mutant, Bc-6-A4, using the silencing vector pCBSilent1-ABA4. The expression levels of *bcaba4* were determined by qRT-PCR and presented as percentage of the value obtained from the parent strain. P: the parent strain Bc-6-A4; R1: a randomly selected transformant whose liquid chromatogram was shown (**c**); R2–R6: five other randomly selected transformants; (**f**) based on the relative ABA productivity to that of the parent strain Bc-6-A4, the 45 randomly selected transformants fell into five categories (0%–20%, 20%–40%, 40%–60%, 60%–80%, and 80%–100% ABA productivity relative to that of the parent strain).

Given the high efficiency of pCBSilent1 in silencing the *eGFP* gene, pCBSilent1-based vectors were used to silence the endogenous gene, *bcaba4*, in an ABA-producing *B. cinerea* mutant, Bc-6-A4 which was derived from *B. cinerea* Bc-6. The sense and antisense sequences of the 774 bp *bcaba4* fragment (without the termination codon) were inserted into the two MCS of pCBSilent1 using the In-Fusion assembly method to generate the RNA silencing vector pCBSilent1-ABA4. The ATMT of the conidia of Bc-6-A4 was also performed to introduce the silencing construct into the genome of this *B. cinerea* strain. After the single conidia of the transformants were regenerated in the presence of 25 μg/mL hygromycin B, DNA extraction, total RNA extraction and cDNA synthesis were performed on 45 randomly selected transformants. Diagnostic PCR using primers PAnoliC-579 and TAntrpC-132 verified the integration of the T-DNA region into the genomes of all these randomly selected transformants. Expression levels of *bcaba4* mRNA in six of the 45 randomly selected transformants were determined by qRT-PCR (Figure 5e). *Bcaba4* mRNA levels in three of the six transformants were reduced to less than 20% of the value obtained from the parent strain Bc-6-A4, which indicated that the expression of *bcaba4* mRNA was dramatically reduced in these transformants compared to that of their parent strain. The single conidia of the transformants and their parent strain, Bc-6-A4, were grown on PDA plates for 15 days. Also the amounts of ABA were determined by HPLC as described above. The ABA titers of all 45 transformants were reduced to some extent (Figure 5f). The absorption peak corresponding to ABA was significantly decreased, similar to that shown in Figure 5c, in 21 out of the 45 transformants. This indicated a significantly decreased ABA productivity (less than 20% relative ABA productivity to that of the parent strain) of these transformants (Figure 5f). While the same assay yielded a significantly higher absorption peak for their parent strain, which indicated that Bc-6-A4 had a stronger ABA productivity. Additionally, the results from the six randomly selected transformants showed a correlation between their ABA productivity and their expression levels of *bcaba4* (Figure 5e).

## 3. Discussion

To uncover the molecular mechanisms underlying secondary metabolite biosynthesis of the filamentous fungus *B. cinerea*, genetic manipulation tools based on high-throughput transformation methods are required to raise, reduce or cancel the expression levels of target genes. Compared to the protoplast-based transformation method with which almost all the gene knock-out studies of *B. cinerea* were based on, the ATMT system is more convenient and does not require any enzymatic treatment processes. The ATMT transformation method transfers part of the tumor-inducing plasmid DNA into the genomes of the recipient fungus. The ATMT of *B. cinerea* was first reported by Rolland *et al.* [33]. Gene disruption [34,35] and the characterization of new pathogenicity-related genes using insertional mutant library construction [19] via ATMT were also reported for *B. cinerea*. The ABA-producing *B. cinerea* strains in our laboratory produce less conidia than the most widely used *B. cinerea* strain B05.10, and few resistant transformed colonies were obtained when performing protoplast-based transformation (data not published), so we adopted the ATMT system to introduce foreign DNA into the genome of these *B. cinerea* strains.

**Figure 6:**
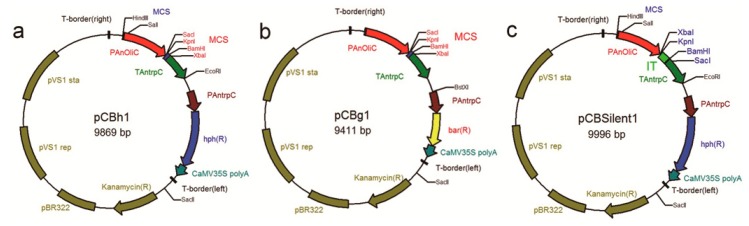
Schematic structures of the *Agrobacterium* vectors. (**a**) The expression vector pCBh1; (**b**) the expression vector pCBg1; (**c**) the RNA silencing vector pCBSilent1. PAnoliC: *Aspergillus nidulans oliC* promoter; PAntrpC: *Aspergillus nidulans trpC* promoter; TAntrpC: *Aspergillus nidulans trpC* terminator; hph (R): hygromycin B phosphotransferase gene conferring resistance to hygromycin B; bar (R): basta-resistance gene conferring resistance to glufosinate; Kanamycin (R): kanamycin resistance gene conferring resistance to kanamycin sulfate; IT: intron 2 of the *Magnaporthe oryzae* cutinase gene.

In this study, two novel expression vectors with different selection markers (hygromycin B for pCBh1 (Figure 6a) and glufosinate for pCBg1 (Figure 6b)) and a silencing vector pCBSilent1 (Figure 6c) were developed to overexpress or silence specific genes in *B. cinerea* via ATMT of this fungus. The In-Fusion assembly method [39,40,41] was adopted for all vector construction experiments in this study. In contrast to the traditional restriction enzyme digestion and ligation method, which is usually limited by a lack of available unique restriction sites in a vector or the target gene, the In-Fusion assembly method, which is ligation-independent, can join any two DNA fragments that have identical 15 bp sequences at their ends. The 15 bp homologous oligonucleotides could be easily introduced by the primers. In this study, the In-Fusion method was adopted to insert a DNA fragment into a restriction enzyme-digested vector. Taking the RNA silencing vector pCBSilent1 as an example, there were just two restriction sites in each of the two MCS (XbaI/KpnI sites for the 5' MCS and BamHI/SacI sites for the 3' MCS), however sense and antisense sequences of a specific gene could be conveniently introduced into this vector to generate pCBSilent1-based silencing vectors that facilitated hairpin RNA generation.

Rolland *et al.* [33] first developed several expression vectors based on ATMT of *B. cinerea* to investigate the transcription activities of the pepstatin-insensitive aspartyl protease ACP1 gene (*bcacp1*) promoter, and the GFP gene or the beta-glucuronidase (GUS)-encoding gene was driven by different versions of the *bcacp1* promoter in these vectors [23,33,42]. In this study, we adopted the widely used strong promoter *A. nidulans oliC* [37,43,44] to trigger the transcription of exogenous or endogenous genes into the genome of *B. cinerea* via ATMT of this fungus. The *eGFP* gene was used to validate the expression efficiency of the two *Agrobacterium* vectors (pCBh1and pCBg1) in *B. cinerea*. The strong GFP fluorescence (Figure 2) in both the conidia and mature aerial hyphae of thetransformants indicated that vectors pCBg1 and pCBh1 could express exogenous genes in *B. cinerea* with high efficiency. An endogenous gene, *bcaba4*, which was suggested to participate in the biosynthesis of ABA in *B. cinerea*, was also overexpressed in the *B. cinerea* wild type strain Bc-6. The expression of *bcaba4* was increased and ABA accumulated dramatically in the transformants (Figure 3). The comparable expression efficiency of the endogenous gene, *bcaba4*, and the exogenous gene, *eGFP*, suggested the consistency and high efficiency of the two expression vectors. Also, the two vectors pCBh1and pCBg1 were constructed with different selection markers: a hygromycin selection marker for pCBh1 and a glufosinate selection marker for pCBg1, which facilitates introducing two genes into the genome of the same recipient *B. cinerea* strain separately.

RNA-mediated gene silencing or RNAi is an important genetic manipulation approach especially for essential genes or multi-copy genes. This gene knock-down strategy results in diminished transcription levels of the target genes and the silencing extent between transformants is usually variable [45]. RNA silencing has emerged as an important strategy for gene function characterization in *B. cinerea*. Patel *et al.* [18] first silenced a superoxide dismutase gene in *B. cinerea*, and several other *B. cinerea* genes have since been silenced using the protoplast-based transformation system: a phospholipase C gene (*bcPLC1*) [21], an argininosuccinate synthase gene (*bcass1*) [20] and a type 2A phosphoprotein phosphatase gene (*bcpp2Ac*) [19]. Rolland *et al.* [23] reported the only RNA silencing study via ATMT of *B. cinerea* in that a putative phospholipase D-encoding gene (*bcpld*) was silenced via this transformation method. In his study, a 300 bp DNA fragment of the *gfp* gene was used as the spacer to generate the hairpin-RNA construct, but the efficiency of their RNA silencing tool was not deciphered. In our study, pCBSilent1, a silencing vector facilitating ATMT of *B. cinerea*, was developed using the 147 bp intron of *M. oryzae* cutinase as the spacer. The *eGFP* gene was used to verify silencing efficiency of this novel *Agrobacterium* vector. Compared to the parent strain *B. cinerea* Bc-6-eGFP, reduced GFP fluorescence, was visualized from all the randomly selected transformants, and 44.4% of them showed almost undetectable fluorescence (Figure 4c). QRT-PCR results showed that *eGFP* mRNA levels in half of the transformants were reduced to less than 20% of the value obtained from their parent strain. These results together indicated that the silencing construct generated from this pCBSilent1-based vector significantly reduced the eGFP expression in these transformants. *Bcaba4* gene was also silenced in the ABA-producing *B. cinerea* mutant Bc-6-A4. QRT-PCR results showed that the expression of *bcaba4* mRNA was dramatically reduced in half of the randomly selected transformants (Figure 5d), and 46.7% of all the randomly selected transformants showed significantly decreased ABA production potential (less than 20% relative ABA productivity to that of the parent strain) (Figure 5f). These results indicated that pCBSilent1-based constructs induced strong silencing in these transformants, which was comparable to the eGFP silencing results. So the consistency of pCBSilent1-based vectors in inducing gene silencing in *B. cinerea* was suggested. The spacer in the RNAi cassette affects the silencing efficiency of the hairpin RNA-expressing strategy. Nakayashiki *et al.* [38] investigated the silencing efficiency of pSilent1-based vectors with three spacers, and the results showed that the silencing vector employing the 147 bp intron of *M. oryzae* cutinase was more efficient in silencing genes than the 542 bp fragment of the GUS gene and the 850 bp intron of *M. oryzae* chitin binding protein. In this study, we also employed the 147 bp intron of *M. oryzae* cutinase as the spacer. The results showed that the hpRNA-expressing strategy with a *M. oryzae* cutinase intron 2 as the spacer was very efficient in silencing genes in *B. cinerea*.

In this study, when *bcaba4* gene was overexpressed in the *B. cinerea* wild type strain Bc-6, moderately high ABA titers were detected (Figure 3). The *bcaba4* silencing results showed that ABA production can be significantly reduced when the *bcaba4* gene was silenced. Together the gene overexpression and RNA silencing results showed a correlation between the expression levels of *bcaba4* and the ABA productivity of *B. cinerea* strains. In a previous study, Siewers *et al.* [26] determined an ABA biosynthetic gene cluster in *B. cinerea* that consists of four genes: two putative P450 monooxygenase-encoding genes (*bcaba1* and *bcaba2*), a gene with unknown function (*bcaba3*), and a short-chain dehydrogenase/reductase (*bcaba4*). Other than the ΔBcaba1, ΔBcaba2, and ΔBcaba3 mutants which contained no residual ABA, the ΔBcaba4 transformants still produced some but apparently less ABA than the control strain [26]. *Bcaba4* gene was hence suggested to participate in the biosynthesis of this SM in *B. cinerea*. Our gene overexpression and RNA silencing results confirmed the participation of the *bcaba4* gene in the ABA biosynthesis of the phytopathogenic ascomycete *B. cinerea* and the assumption that *bcaba4* is a limiting factor for ABA biosynthesis could also be put forward. This study showed that the overexpression of *bcaba4* gene can promote ABA production and this is the first report to use an RNA silencing vector to manipulate a SM biosynthetic gene in *B. cinerea*, to our knowledge.

As an important phytohormone, the sesquiterpene ABA is economically valuable and several *B. cinerea* strains have been used for fermentative ABA production at the industrial scale [10,11]. The vectors developed in this study will be applied to raise the transcriptional levels of ABA biosynthetic genes and to reduce the expression of the key biosynthetic genes of relevant terpenes that are synthesized from the same precursor with ABA. Additionally, these genetic manipulations will facilitate the construction of genetically engineered ABA-producing *B. cinerea* strains.

These newly developed reverse genetic tools that facilitate gene function characterization in *B. cinerea* have already been used to raise or reduce gene expression levels of the most widely used *B. cinerea* strains B05.10 and T4 (data not published) and may also be used in other filamentous fungi. The three *Agrobacterium* binary vectors we developed in this study still have potential to be further improved using the In-Fusion method. For example, additional MCS modules could be introduced to both sides of the two existing cassettes in the three vectors in order to insert 5'- and 3'-flanking regions of a specific gene; and the resulting vectors could be used for targeted integration into a defined locus of the *B. cinerea* genome. Such transformants obtained via gene overexpression or RNA silencing manipulations would have identical genomic backgrounds.

## 4. Experimental Section

### 4.1. Strains, Media, and ATMT

The *B. cinerea* wild type strain Bc-6 [36] was isolated from stems and leaves of wheat in southwest China. The eGFP-expressing *B. cinerea* mutant, Bc-6-eGFP and the ABA-producing *B. cinerea* mutant, Bc-6-A4 were derived from *B. cinerea* Bc-6. *Agrobacterium tumefaciens* strain EHA105 [46] was used to transform *B. cinerea* strains. *Escherichia coli* strain DH5ɑ was used as the host for transformation and genetic manipulation of plasmid DNA [47]. 50 μg/mL kanamycin (Amresco, Solon, OH, USA) was used to select positive colonies on LB plates. *B. cinerea* strains were grown on potato dextrose agar (PDA) slants at 26 °C for 7 days. After spore maturation, ATMT was performed as described by Rolland *et al.* [33] with some modifications (data not published). Twenty five μg/mL hygromycin B (Sigma, St. Louis, MO, USA) and 25 μg/mL glufosinate-ammonium (Sigma) were used to select transformants on solid PDA plates. Further selection of gene overexpression or RNA silencing mutants was performed by regenerating single conidia of the transformants on PDA plates supplemented with corresponding antibiotics.

### 4.2. Construction of the Gene Overexpression Binary Vector pCBh1

All the primers used in this study were listed in Table 1. For pCBh1 construction, the *Aspergillus nidulans oliC* promoter was amplified using the plasmid pLOB7 [37], provided by J. van Kan (Wageningen University and Research Centre), as the template with primer pair pUC19-AnoliC and pUC19-PstI-AnoliC. The reverse primer pUC19-PstI-AnoliC used here contains additional 45 bp oligonucleotides homologous to the PstI-EcoRI fragment of multiple cloning sites in the plasmid pUC19. The *Aspergillus nidulans trpC* terminator was also amplified from pLOB7 with primer pair pUC19-BamHI-AntrpC and pUC19-AntrpC. The forward primer pUC19-BamHI-AntrpC used here contains additional 36 bp oligonucleotides homologous to the BamHI-EcoRI fragment of multiple cloning sites in the plasmid pUC19. The two PCR products were connected with overlap extension PCR to generate the expression cassette that consisted of three modules: the *Aspergillus nidulans oliC* promoter, a multiple cloning site identical to that of pUC19 and the *Aspergillus nidulans trpC* terminator. The whole expression cassette was then amplified with primer pair p1300-AnoliC and p1300-AntrpC. The forward primer p1300-AnoliC has additional 15 bp oligonucleotides identical to the 15 bp DNA sequence including HindIII restriction site and adjacent to (excluding) the SbfI restriction site of the MCS in the vector pBHt2 [30], provided by Prof. Seogchan Kang (The Pennsylvania State University, University Park, PA, USA). The reverse primer p1300-AntrpC also has 15 bp identical to the DNA sequence including EcoRI restriction site and adjacent to the SacI restriction site of the MCS in the plasmid pBHt2. In-Fusion assembly reactions (using the Clontech In-Fusion PCR Cloning Kit, Mountain View, CA, USA) [39,40,41] were performed with this PCR fragment and SbfI/SacI linearized pBHt2 and the final 9869 bp vector pCBh1 for gene overexpression was generated. DNA sequencing was performed to all the modules used during the construction procedure to verify their sequence.

### 4.3. Construction of the Gene Overexpression Binary Vector pCBg1

To construct the basta-resistance cassette for pCBg1, the coding domain sequence (CDS) of the basta-resistance (bar) gene was amplified using the plasmid pBARKS1 [48] as the template with the primer pair bar-5 and bar-3. The reverse primer bar-3 contains 53 bp oligonucleotides, and the KpnI restriction site near the termination codon of the bar gene was replaced with this primer via site-directed mutagenesis without any alteration in its protein sequence. The *Aspergillus nidulans trpC* promoter was amplified using the plasmid pBHt2 as the template with the primer pair BstXI-PAntrpC-5 and PAntrpC-3. The forward primer BstXI-PAntrpC-5 contains the additional 15 bp oligonucleotides identical to the 15 bp DNA sequence after the EcoRI restriction site and adjacent to the unique BstXI site of the binary vector pCAMBIA-1300-221 (available online: http://www.cambia.org.au). The CaMV35S polyA module in the plasmid pCAMBIA-1300-221 was selected as the terminator for the basta-resistance cassette, and the entire sequence from the first base immediately after the hygromycin module to the unique SacII restriction site of the plasmid pCAMBIA-1300-221 was amplified with the primer pair CaMV35S-5 and SacII-leftborder-3 to produce one PCR product. The reverse primer SacII-leftborder-3 also contains 15 bp identical to the DNA sequence after (and excluding) the SacII site of the plasmid pCAMBIA-1300-221. The above three PCR products were connected with overlap extension PCR to generate the basta-resistance cassette, which consisted of three modules: the *Aspergillus nidulans trpC* promoter, the CDS of the bar gene and the CaMV35S polyA terminator. In-Fusion assembly reactions were performed with this amplified basta-resistance fragment, BstXI/SacII linearized pCAMBIA-1300-221 and an intermediate vector was generated.

To construct the final expression vector pCBg1, the expression cassette was amplified using the sequence-verified pCBh1 as the template with the primer pair p1300-HindIII and p1300-EcoRI. The primers also contain their corresponding 15 bp homologous oligonucleotides, which are identical to the DNA sequences adjacent to the insertion sites in the targeting vector (the intermediate vector constructed above in this case). After sequence verification, the intermediate vector was linearized with HindIII/EcoRI, and In-Fusion assembly reactions were performed with this linearized intermediate vector and the PCR amplified expression cassette to generate the final 9411 bp expression vector, pCBg1. This binary vector contains two cassettes in its T-DNA region: an expression cassette for the expression of a specific target gene and a basta-resistance cassette, which acts as the positive selection marker and confers resistance to glufosinate.

### 4.4. Construction of the RNA Silencing Binary Vector pCBSilent1

The RNA silencing vector pCBSilent1 was constructed based on the expression vector pCBh1 and the RNA silencing vector pSilent-1 [38], which was developed by Prof. Hitoshi Nakayashiki (Kobe University, Kobe, Japan); we obtained this silencing vector from the Fungal Genetics Stock Center (available online; www.fgsc.net). Intron 2 of the cutinase (CUT) gene from the rice blast fungus *Magnaporthe oryzae* was used as the spacer for hairpin RNA expression and was amplified from the plasmid pSilent-1 with the primer pair 5-AnoliC-CUT and 3-AntrpC-CUT. The forward primer, 5-AnoliC-CUT, contains an additional 15 bp oligonucleotides identical to the last 15 bp of the *A. nidulans oliC* promoter in the expression cassette of the binary vector pCBh1, and the reverse primer, 3-AntrpC-CUT, contains an additional 15 bp identical to the first 15 bp of the *A. nidulans trpC* terminator in the same expression cassette. The PCR amplification conducted here was also a restriction site-generating PCR, which introduced the XbaI and KpnI restriction sites via the forward primer, while BamHI and SacI restriction sites were introduced with the reverse primer. In-Fusion assembly reactions were performed with this PCR product and SbfI/SacI linearized plasmid pCBh1 to generate the final 9996 bp RNA silencing vector, pCBSilent1, for ATMT. To construct the RNA silencing vector for a specific target gene, the gene sequence was inserted into both the 5' multiple cloning site and the 3' multiple cloning site, separated by the spacer in pCBSilent1. The transcriptional unit was organized as follows: *A. nidulans oliC* promoter, sense sequence of the target gene, spacer, antisense of the target gene and *A. nidulans trpC* terminator, and this organization facilitates hairpin RNA expression.

### 4.5. Overexpression and Gene Silencing of eGFP in B. cinerea Strains

The binary vectors pCBh1 and pCBg1 were used to introduce eGFP into the genome of *B. cinerea* wild type strain Bc-6. The eGFP CDS was amplified using the plasmid pEGFP-N1 (Clontech) as the template with the primer pair AnoliC-eGFP and AntrpC-eGFP. The first 15 bp oligonucleotides of the forward primer are homologous to the ending sequence of the promoter *oliC*, which is adjacent to multiple cloning sites of the expression cassette. The first 15 bp of the reverse primer are also homologous to the starting sequence of the terminator *trpC*. In-Fusion assembly reactions were performed with this PCR product and SbfI/SacI linearized pCBh1 or HindIII/EcoRI linearized pCBg1 to generate eGFP overexpression vectors (named pCBh1-eGFP and pCBg1-eGFP). After sequence verification, the two vectors were used for the ATMT of the conidia of *B. cinerea* wild type strain Bc-6. Hygromycin B (25 μg/mL) or glufosinate-ammonium (25 μg/mL) were used to select transformants. Single conidia of the transformants were regenerated in the presence of corresponding antibiotics to further select the transformants.

The RNA silencing vector pCBSilent1-eGFP based on pCBSilent1 was used to silence the eGFP gene in an eGFP-expressing *B. cinerea* strain, Bc-6-eGFP. The entire sense sequence of the eGFP gene (without the termination codon) was amplified and inserted into the XbaI/KpnI restriction sites of pCBSilent1 by means of In-Fusion assembly reactions to generate an intermediate vector. The entire antisense sequence of eGFP (without antisense oligonucleotides of the termination codon) was then amplified and inserted into the BamHI/SacI sites of the intermediate vector with In-Fusion reactions to generate the final RNA silencing vector, pCBSilent1-eGFP. After sequence verification, the plasmid pCBSilent1-eGFP was used for the ATMT of the conidia of the eGFP-expressing *B. cinerea* strain, Bc-6-eGFP. Hygromycin B (25 μg/mL) was used to select transformants, and single conidia of the transformants were regenerated in the presence of hygromycin B to further select transformants.

### 4.6. Imaging of GFP Fluorescence

The eGFP fluorescent images of the conidia and mature aerial hyphae of the selected transformants were captured on a Zeiss Axioplan 2 fluorescence microscope (ZEISS, Oberkochen, Germany) equipped with a CCD camera (ZEISS) and visualized with the AxioVision software (ZEISS). The fluorescent images from different *B. cinerea* strains were obtained with the same exposure times and instrument parameters.

### 4.7. Overexpression and Gene Silencing of Bcaba4 Gene in B. cinerea Strains

The gene overexpression vector for the *bcaba4* gene was constructed based on pCBg1. The *bcaba4* CDS was amplified using the cDNA of the *B. cinerea* wild type strain Bc-6 as the template with the primer pair AnoliC-ABA4 and AntrpC-ABA4. The primers also contained corresponding 15 bp homologous oligonucleotides that are identical to the DNA sequences adjacent to the MCS of the targeting vector, pCBg1. In-Fusion reactions were performed with the PCR product and the HindIII/EcoRI linearized pCBg1 to generate the expression vector pCBg1-ABA4 with a glufosinate selection marker. After sequence verification, ATMT was performed with pCBg1-ABA4 and the conidia of the *B. cinerea* wild type strain Bc-6. Glufosinate-ammonium (25 μg/mL) was used to select transformants. Single conidia of the transformants were regenerated in the presence of glufosinate-ammonium. To determine whether the *bcaba4* gene had integrated into the genome of the *B. cinerea* mutant Bc-6-A4, the fungal genomic DNA of selected transformants was analyzed with diagnostic PCR using the primer pair PAnoliC-579 and TAntrpC-132.

To construct the RNA silencing vector pCBSilent1-ABA4, the entire sense sequence of the *bcaba4* gene (excluding the termination codon) was amplified and inserted into the XbaI/KpnI sites of pCBSilent1 using In-Fusion reactions to generate an intermediate vector. The entire antisense sequence of *bcaba4* (without antisense oligonucleotides of the termination codon) was then amplified and inserted into the BamHI/SacI sites of the intermediate vector with In-Fusion reactions to generate the RNA silencing vector pCBSilent1-ABA4, which was used to silence the *bcaba4* gene in an ABA-producing *B. cinerea* mutant, Bc-6-A4. After sequence verification, ATMT was performed with pCBSilent1-ABA4 and the conidia of the *B. cinerea* mutant Bc-6-A4. Hygromycin B (25 μg/mL) was used to select transformants, and single conidia of the transformants were further regenerated in the presence of hygromycin B to select the transformants. Diagnostic PCR was also performed with the primer pair PAnoliC-579 and TAntrpC-132 to determine whether the transcriptional unit for hairpin RNA generation had integrated into the genome of the selected transformants.

### 4.8. Extracellular ABA Determination

Single conidia of the *B. cinerea* transformants and their control strains were grown on solid PDA plates for 15 days at 26 °C. The extracellular ABA that was secreted into the PDA medium was extracted with acetone, and the ABA contents of these extracted samples were measured with high performance liquid chromatography (HPLC) using a commercial *S*-(+)-ABA (98% *w*/*w*, Lomon Bio Technology Co., Ltd., Sichuan, China) as the standard sample. The Agilent 1200 Pure Liquid Chromatography system (An Agilent 1260 Infinity Quaternary Pump VL with An Agilent 1260 Infinity Standard Autosampler and an Agilent 1260 Infinity Variable Wavelength Detector) was used with a Luna^®^ 5 µm C18(2) LC Column (Phenomenex, Torrance, CA, USA, Cat # 00G-4252-E0). The acetone-extracted samples were diluted to the same volume, and the ABA counts of these samples were determined based on their absorption at 254 nm. The ABA counts of these extracted samples were also determined with a plant hormone abscisic acid ELISA kit (CUSABIO, Seattle, WA, USA, Cat # CSB-E09159PI) following the manufacturer’s instructions. All measurements were performed independently in triplicate.

### 4.9. qRT-PCR Analysis

Mycelium was collected from the transformants and their control strains, quenched in liquid nitrogen and ground into powder immediately. Total RNA extraction was performed with E.Z.N.A.™ Fungal RNA Miniprep Kit (OMEGA, Tokyo, Japan, Cat # R6840-01) and on-membrane DNase I digestion was performed with E.Z.N.A. RNase-Free DNase I Set (OMEGA, Cat # E1091). Integrity of the extracted RNAs was determined by NanoDrop spectrophotometer (Thermo Fisher Scientific, Waltham, MA, USA) and agarose gel electrophoresis. First-strand cDNA was synthesized from 1 µg total RNA using ReverTra Ace-ɑ-^®^ kit (Cat # FSK-101, TOYOBO, Satte City, Japan) with the Oligo(dT)20 primer. The synthesized cDNA was used as the template for PCR amplification of *bcaba4* gene (BC1G_07529) with the primer pair RT-ABA4-5/RT-ABA4-3 and *eGFP* gene with the primer pair RT-gfp-5/RT-gfp-3. The *B. cinerea* tubulin gene (BC1G_05600) [49] was used to correct for sample-to-sample variation in the amount of RNA (with the primer pair RT-tubA-5/RT-tubA-3). Amplification was carried out in the CFX96 Real-Time PCR Detection System (BioRad, Hercules, CA, USA), with the TransStart Green qPCR SuperMix UDG (Transgen, Beijing, China). The relative mRNA amounts were calculated by the 2^−ΔΔ*C*t^ method from the mean of three independent determinations of the threshold cycle [50].

## 5. Conclusions

In conclusion, two novel gene expression vectors (pCBh1 and pCBg1) with different selection markers and one novel RNA silencing vector, pCBSilent1, were developed in this work to facilitate genetic manipulation of the filamentous fungus *B cinerea* via the ATMT method. The In-Fusion assembly method, which is ligation-independent and can join any two DNA fragments that have identical 15 bp sequences at their ends, was adopted for all the vector construction experiments in this study. For the first time, a strong promoter was adopted to trigger the transcription of exogenous or endogenous genes into the genome of *B. cinerea* via ATMT of this fungus. An exogenous gene, *eGFP*, and an endogenous gene, *bcaba4*, which was suggested to participate in the biosynthesis of ABA in *B. cinerea*, were used to validate the working efficiency of the reverse genetic tools. The results indicated high expression efficiency or silencing efficiency of these newly developed genetic manipulation tools.

The gene overexpression and RNA silencing results confirmed the participation of the *bcaba4* gene in the ABA biosynthesis of *B. cinerea*. Also for the first time, this study showed that the overexpression of *bcaba4* gene can promote ABA production. The vectors developed in this study could facilitate the construction of genetically engineered ABA-producing *B. cinerea* strains. These reverse genetic tools have already been used to raise or reduce gene expression levels of the most widely used *B. cinerea* strains B05.10 and T4 and may also be used in other filamentous fungi.
